# Teleassistance for Patients With Type 1 Diabetes During the COVID-19 Pandemic: Results of a Pilot Study

**DOI:** 10.2196/24552

**Published:** 2021-04-06

**Authors:** Martina Parise, Linda Tartaglione, Antonio Cutruzzolà, Maria Ida Maiorino, Katherine Esposito, Dario Pitocco, Agostino Gnasso, Concetta Irace

**Affiliations:** 1 Department of Experimental and Clinical Medicine University Magna Graecia Catanzaro Catanzaro Italy; 2 IRCCS University Agostino Gemelli Rome Italy; 3 Department of Advanced Medical and Surgical Science University Vanvitelli, Napoli Naples Italy; 4 Department of Advanced Medical and Surgical Science University Magna Graecia Catanzaro Naples Italy; 5 Department of Health Science University Magna Graecia Catanzaro Catanzaro Italy

**Keywords:** chronic disease, COVID-19, diabetes, effectiveness, management, technology, teleassistance, telehealth, telemedicine, time in range, type 1 diabetes

## Abstract

**Background:**

Telemedicine use in chronic disease management has markedly increased during health emergencies due to COVID-19. Diabetes and technologies supporting diabetes care, including glucose monitoring devices, software analyzing glucose data, and insulin delivering systems, would facilitate remote and structured disease management. Indeed, most of the currently available technologies to store and transfer web-based data to be shared with health care providers.

**Objective:**

During the COVID-19 pandemic, we provided our patients the opportunity to manage their diabetes remotely by implementing technology. Therefore, this study aimed to evaluate the effectiveness of 2 virtual visits on glycemic control parameters among patients with type 1 diabetes (T1D) during the lockdown period.

**Methods:**

This prospective observational study included T1D patients who completed 2 virtual visits during the lockdown period. The glucose outcomes that reflected the benefits of the virtual consultation were time in range (TIR), time above range, time below range, mean daily glucose, glucose management indicator (GMI), and glycemic variability. This metric was generated using specific computer programs that automatically upload data from the devices used to monitor blood or interstitial glucose levels. If needed, we changed the ongoing treatment at the first virtual visit.

**Results:**

Among 209 eligible patients with T1D, 166 completed 2 virtual visits, 35 failed to download glucose data, and 8 declined the visit. Among the patients not included in the study, we observed a significantly lower proportion of continuous glucose monitoring (CGM) and continuous subcutaneous insulin infusion (CSII) users (n=7/43, 16% vs n=155/166, 93.4% and n=9/43, 21% vs n=128/166, 77.1%, respectively; *P*<.001) compared to patients who completed the study. TIR significantly increased from the first (62%, SD 18%) to the second (65%, SD 16%) virtual visit (*P*=.02); this increase was more marked among patients using the traditional meter (n=11; baseline TIR=55%, SD 17% and follow-up TIR=66%, SD 13%; *P*=.01) than among those using CGM, and in those with a baseline GMI of ≥7.5% (n=46; baseline TIR=45%, SD 15% and follow-up TIR=53%, SD 18%; *P*<.001) than in those with a GMI of <7.5% (n=120; baseline TIR=68%, SD 15% and follow-up TIR=69%, SD 15%; *P*=.98). The only variable independently associated with TIR was the change of ongoing therapy. The unstandardized beta coefficient (B) and 95% CI were 5 (95% CI 0.7-8.0) (*P*=.02). The type of glucose monitoring device and insulin delivery systems did not influence glucometric parameters.

**Conclusions:**

These findings indicate that the structured virtual visits help maintain and improve glycemic control in situations where in-person visits are not feasible.

## Introduction

### Background

The COVID-19 pandemic has restricted access to medical clinics in order to prevent the risk of infection. In this context, routine care for patients with chronic diseases such as diabetes has emerged as a major challenge. Several strategies have been implemented to support patients with type 1 diabetes (T1D) and type 2 diabetes during the COVID-19 pandemic and to provide adequate assistance to avoid disease exacerbation [1-4]. Patients with diabetes have been recommended to follow general guidelines on infection risk reduction, blood glucose monitoring, taking medication, injecting insulin and noninsulin drugs adequately, and maintaining a healthy lifestyle (through diet and physical activity) [5].

Furthermore, experts have strongly suggested implementing the practice of downloading glucose data with dedicated software and sharing information with the health care providers to facilitate the provision of remote assistance [6]. More generally, the efficacy of virtual visits through telephone calls, text messages, mobile apps, or electronic visits—outside the context of the COVID-19 pandemic—has been described in some studies and systematic reviews. The current evidence is not univocal, and text messages seem more effective than web-based interventions [7-10]. Despite the number of published articles reporting a consensus on video consultations and guidelines for managing diabetes in the COVID-19 setting, no prospective evidence is available regarding the efficacy of teleassistance as an alternative to ambulatory visits during the pandemic [1,2,5,6]. Indeed, most studies, with a relevant number of patients, are retrospective and have analyzed data remotely with specific software that download glucose data without contacting the patients.

### Aim and Hypothesis

As a necessary measure, we have implemented remote medical examination of patients with T1D during the lockdown period (March 10 to June 3, 2020). In this study, we assessed new glucometric parameters collected during 2 different virtual visits. We hypothesized that the COVID-19 pandemic might affect metabolic control in patients with diabetes owing to limited access to diabetes care centers and poor compliance to lifestyle recommendations. We accordingly designed a pilot study to prospectively analyze the effectiveness of 2 structured virtual visits (video or telephone consultations) on the basis of the time in range (TIR), time above range (TAR), time below range (TBR), mean daily glucose, glucose management indicator (GMI), and glycemic variability [11].

## Methods

### Patients

Our pilot study is a prospective, single-arm, observational study including patients with T1D who completed 2 virtual visits (baseline and follow-up visits) from the start (March 10, 2020) to the end (June 3, 2020) of the lockdown period. The protocol was preliminarily submitted and approved by the local Ethical Committee “Regione Calabria, Area Centro” (approval# 79-2020). In total, 3 diabetes care centers at teaching hospitals were involved in this study. A nurse or physician contacted the patients through the telephone before the first virtual visit (first contact) to explain the study's purpose to them. Patients who provided verbal consent were enrolled. Baseline characteristics of patients who declined to participate in the study were collected and compared to those of patients who agreed to participate. During the telephone call, patients were informed about or instructed, if necessary, on how to download the data from continuous glucose monitoring (CGM) systems or a traditional meter into the cloud by using specific software, such as Clarity, Care Link, Eversense diabetes management system (DMS), Accu-Chek Connect DMS, and share the data through the cloud or email. They were also invited to send recent laboratory findings or reports from other specialists via email or mobile apps. Patients using traditional meters were strongly suggested to check their blood glucose levels before each meal and in the presence of symptoms suggestive of hypoglycemia. During the second contact (baseline virtual visit), the physician verified the clinical conditions, ongoing treatment, and adherence to healthy lifestyle recommendations. Blood glucose data were monitored, and treatment was changed accordingly. The second virtual follow-up visit (third contact) was decided on the basis of clinical conditions and glycemic control and completed as the baseline visit. All information collected during the virtual visit were uploaded in the electronic medical files, and the summary of the visits was shared via email.

### Data Collection

For the present analysis, the following parameters were collected: TIR, TAR, TBR, and mean (SD) daily glucose levels determined 2 weeks before the baseline and follow-up visits. The coefficient of variation (CV)—an estimate of glycemic variability—and the GMI—an estimate of glycated hemoglobin levels—were determined using the following formulae if not automatically generated by the software analyzing glucose data [12]:

CV (%) = [(SD/Mean Glucose) × 100] **(1)**

GMI (%) = 3.38 + 0.02345 × (Mean glucose in mg/dL) **(2)**

### Statistical Analyses

Statistical analyses were performed using SPSS for Macintosh (version 23, IBM Corp). Patients who declined to participate or complete 2 virtual visits were excluded from the analyses. For the statistical analyses, patients were grouped as follows: those included and excluded from the study, those using the sensor and injecting insulin with a pump (CGM + continuous subcutaneous insulin infusion [CSII]), those using the sensor and with multiple daily insulin injection (CGM+MDI), and those testing blood glucose with a traditional meter (ie, self-monitoring blood glucose [SMBG]) and injecting insulin with a pump or through MDI (CSII or MDI+SMBG). Sensor users included patients using intermittently scanned CGM (isCGM) and real-time CGM (rtCGM). Variables including baseline mean daily glucose, GMI, and TBR were not normally distributed. Parametric and nonparametric tests were performed accordingly.

The 2-tailed *t* test for unpaired data and the Mann-Whitney *U* test were used to compare between-group differences. The 2-tailed *t* test for paired data and the Wilcoxon Signed-Rank test were used to compare variables between baseline and follow-up visits. The chi-square test was used to compare percentages (CGM or sensor use and MDI or CSII treatment) between groups. Multivariable regression analysis was performed to evaluate variables independently associated with the absolute difference in TIR between baseline and follow-up visits. Independent variables included in the model were age, disease duration, use of the sensor or pump, and therapy change. The absolute difference in TIR, calculated as the difference between the follow-up TIR and the baseline TIR, was not normally distributed and hence log-transformed before regression analysis.

## Results

### Characteristics of Patients

In total, 209 patients were scheduled for in-person visits during the lockdown, from March 10 to June 3, 2020. Of them, 43 (20.6%) patients declined to participate in the study. Furthermore, 35 (16.7%) patients stated difficulties in downloading or sharing data via email, and 8 (3.8%) did not attend the virtual visit. The remaining 166 (79.4%) patients were enrolled and assessed. The average mean interval between the 2 virtual visits was 11 weeks. The characteristics of patients included and excluded from the study are summarized in Table 1. The proportion of CGM and CSII users was significantly higher among included patients than among excluded patients (n=155/166, 93.4% vs n=7/43, 16%, respectively; *P*<.001). Among 166 patients included in this study, 11 (6.6%) monitored their blood glucose levels with SMBG, 20 (12.0%) used the isCGM, and 135 (81.3%) used rtCGM. Among the excluded 43 patients, only 7 (16%) were using CGM, while the remaining 36 (84%) were using SMBG. All patients monitoring blood glucose through the traditional meter used the Accu-Chek Connect DMS. All the patients included in the study had strips and sensors to monitor their glucose levels during the lockdown. The mean number of tests per day was 4.4 [SD 1.9] and 5.1 [SD 2.4] (*P*=.21) among patients using SMBG and 9.5 [SD 4.9] and 9.8 [SD 6.1] (*P*=.70) among those using isCGM at baseline and follow-up visits, respectively.

**Table 1:** Characteristics of patients with type 1 diabetes included and excluded from the analyses (N=209).

Variables	Patients included (n=166)	Patients excluded (n=43)	*P* value
Age (years), mean (SD)	40 (14)	37 (15)	N/A^a^
Males, n (%)	80 (48.2)	21 (49)	N/A
Disease duration (years), mean (SD)	20 (11)	17 (9)	N/A
SMBG^b^ users, n (%)	11 (6.6)	36 (84)	<.001
CGM^c^ (isCGM^d^ + rtCGM^e^) users, n (%)	155 (93.4)	7 (16)	<.001
MDI^f^ users, n (%)	38 (22.9)	34 (79)	<.001
CSII^g^ users, n (%)	128 (77.1)	9 (21)	<.001

^a^N/A: not applicable.

^b^SMBG: self-monitoring blood glucose.

^c^CGM: continuous glucose monitoring.

^d^isCGM: intermittently scanned continuous glucose monitoring.

^e^rtCGM: real-time continuous glucose monitoring.

^f^MDI: multiple daily insulin injection.

^g^CSII: continuous subcutaneous insulin infusion.

### Glucometric Characteristics at Baseline and Follow-up Visits

We compared glucometric parameters measured at the baseline and follow-up visits among all patients included in the study and grouped in accordance with different combinations of insulin delivery methods and glucose monitoring systems (CSII+CGM, MDI+CGM, and CSII or MDI+SMBG) (Table 2). The TIR significantly increased from the baseline to the follow-up visit in all patients with T1D (62%, SD 18% vs 65%, SD 16%, respectively; *P*=.02) and in the CSII or MDI+SMBG group (55%, SD 17% vs 66%, SD 13%, respectively; *P*=.01). Furthermore, the CSII or MDI+SMBG group displayed a significant improvement in the TAR at baseline and follow-up visits (40%, SD 18% vs 28%, SD 15%, respectively; *P*=.03), mean daily glucose (176 [SD 49] mg/dL vs 150 [SD 25] mg/dL; *P*=.04), GMI (7.5%, SD 1.1% vs 6.9% SD 0.6%; *P*=.04), and CV (36%, SD 8% vs 42%, SD 9%; *P*=.04) compared to the other groups.

Based on the baseline GMI findings among all patients included in the study, we observed that the TIR significantly improved from baseline to follow-up visits among those with a GMI of ≥7.5% (n=46; 45%, SD 15% vs 53%, SD 18%; *P*<.01) compared to those with a GMI of <7.5% (n=120; 68%, SD 15% vs 69%, SD 15%; *P*=.98).

**Table 2:** Glucometric parameters at baseline and follow-up virtual visits in all patients and in those grouped in accordance with the insulin delivery method and glucose monitoring system (N=166).

Patient groups		Parameters		*P* value^a^
		Baseline visit	Follow-up visit	
**Time in range (%), mean (SD)**				
	All patients (n=166)	62 (18)	65 (16)	*.02*
	CSII^b^+CGM^c^ (n=122)	63 (17)	65 (17)	.24
	MDI^d^+CGM (n=33)	62 (19)	64 (17)	.19
	CSII or MDI+SMBG^e^ (n=11)	55 (17)	66 (13)	*.01*
**Time below range (%), mean (SD)**				
	All patients (n=166)	3.5 (4.1)	3.4 (3.8)	.58
	CSII+CGM (n=122)	3.2 (4.0)	3.1 (3.7)	.86
	MDI+CGM (n=33)	4.4 (4.3)	3.7 (3.3)	.34
	CSII or MDI+SMBG (n=11)	4.7 (4.0)	5.8 (5.0)	.33
**Time above range (%), mean (SD)**				
	All patients (n=166)	34 (18)	32 (18)	.08
	CSII+CGM (n=122)	34 (18)	33 (18)	.40
	MDI+CGM (n=33)	33 (21)	32 (18)	.52
	CSII or MDI+SMBG (n=11)	40 (18)	28 (15)	*.03*
**Mean daily glucose (mg/dL), mean (SD)**				
	All patients (n=166)	163 (29)	159 (25)	.25
	CSII+CGM (n=122)	162 (25)	161 (24)	.90
	MDI+CGM (n=33)	162 (37)	157 (26)	.17
	CSII or MDI+SMBG (n=11)	176 (49)	150 (25)	*.04*
**Coefficient of variation (%), mean (SD)**				
	All patients (n=166)	34 (6)	34 (7)	.32
	CSII+CGM (n=122)	34 (6)	33 (7)	.93
	MDI+CGM (n=33)	36 (7)	35 (7)	.55
	CSII or MDI+SMBG (n=11)	36 (8)	42 (9)	*.04*
**Glucose management indicator (%), mean (SD)**				
	All patients (n=166)	7.2 (0.7)	7.1 (0.6)	.23
	CSII+CGM (n=122)	7.2 (0.6)	7.1 (0.6)	.90
	MDI+CGM (n=33)	7.2 (0.8)	7.0 (0.6)	.12
	CSII or MDI+SMBG (n=11)	7.5 (1.1)	6.9 (0.6)	*.04*

^a^Significant *P* values are shown in italics.

^b^CSII: continuous subcutaneous insulin infusion.

^c^CGM: continuous glucose monitoring.

^d^MDI: multiple daily insulin injection.

^e^SMBG: self-monitoring blood glucose.

In total, 104 (63%) patients were suggested a change of therapy during the baseline visit. Among them, 97 (93%) used CGM (isCGM or rtCGM), and 84 (81%) used CSII. These proportions were comparable with those of patients who were not suggested a change of therapy (CGM: 93%; *P*=.60 and CSII: 71%; *P*=.10). The absolute difference in TIR between baseline and follow-up visits was significantly higher among patients who were suggested a changed of therapy (4%, SD 10%) than among those who were not suggested a change of therapy (0.1%, SD 10%) (*P*=.04). No significant difference was observed in the TBR, TAR, mean daily glucose, CV, and GMI between these 2 groups (data not shown).

None of the patients had diabetic ketoacidosis or severe hypoglycemia requiring hospitalization in the interval between the 2 virtual visits.

Multivariable regression analysis revealed that the change of therapy was the only variable independently associated with the absolute difference in TIR between baseline and follow-up visits. The unstandardized beta coefficient (B) and 95% CI were 5 (95% CI 0.7-8.0) (*P*=.02).

## Discussion

### Principal Findings

Our study shows that the structured virtual visits adequately maintain pre-existing glycemic control or improve the time spent in the target range among individuals with T1D during an emergency when in-person consultations are not permitted.

The benefits of virtual visits were discernible among patients using the traditional meters regardless of the type of insulin delivery modality (MDI or CSII) and among patients with a baseline GMI of ≥7.5%. These results suggest certain considerations. The telephone call preceding the baseline virtual visit may have encouraged patients using SMBG to download data from the meter into diabetes management systems, thus facilitating the subsequent interpretation of glucose data. In the actual scenario, it is common for patients using SMBG to not bring the meter along with them during in-person visits or to not have devices capable of connecting to the cloud, limiting appropriate adjustment of treatment. Patients using SMBG had higher values of GMI and a lower TIR, despite not presenting significant findings, than sensor users, which may have induced physicians to strengthen the more the general suggestions for diabetes management and convince patients to maintain a healthy lifestyle rather have a change of therapy. It could be argued that new metrics have been developed for glucose data collected by the sensor and that our results obtained from patients using the traditional meter might be due to chance. However, we have recently demonstrated that a strict correlation between TIR calculated by specific software, after downloading SMBG values, is significantly correlated with glycated hemoglobin levels. The strength of this association in our study was comparable to that between TIR calculated from CGM and glycated hemoglobin levels reported in other studies [13]. Patients using the meter reported a significant increase in CV compared to the other groups. This result can be explained by the higher daily fluctuation in the number of SMBG users compared to that of CGM users. Indeed, at the follow-up visit, mean glucose levels decreased by 11%, and the CV increased by 2.9%. This might be an unfavorable finding, if sustained in the long term. Some of the aforementioned considerations also apply to patients using CGM who had not experienced an exacerbation of glucose levels during the lockdown.

Patients with a GMI of ≥7.5% may have better managed their diabetes during the pandemic and may have had a healthy lifestyle. We believe that patients generally have had much more time available, owing to restrictions associated with stay-at-home orders. This may have stimulated a more in-depth analysis of glycemic data and potential interventions on incorrect habits.

Patients using the sensor or wearing the pump did not experience an exacerbation in glucose levels during the lockdown. In our opinion, this is a remarkable finding, given the current state of emergency. Sensor and pump users are in general well-educated and motivated to check and self-manage the disease. It is noteworthy that the proportion of pump and sensor users was very low among patients excluded from the study. The main limitation associated with patient recruitment was problems faced with downloading the glucose data.

Multivariable regression analysis revealed that a change of therapy during the virtual visit was the only variable independently associated with the absolute difference in TIR. Insulin therapy can be safely modified during a structured virtual visit. Indeed, none of the patients included in the teleassistance had a severe hypoglycemic episode or ketoacidosis.

Our results reinforce the evidence that virtual consultations may lead to appropriate care during a pandemic or when an in-person visit cannot be performed for any reason.

### Comparison With Previous Studies

Recent case studies have reported that teleassistance can be provided safely and effectively for new-onset T1D and ketoacidosis, thus preventing hospital admission [14,15].

A retrospective study, including 13 adolescent patients with T1D, has reported that physical activity regularly performed during lockdown is associated with an improvement in the TIR [16]. Unfortunately, we did not collect information on physical activity from our patient cohort. However, we speculate that the patients included in our study stayed active at home in some way.

Another retrospective study, including 92 patients with T1D who use CGM systems, has reported an increase in the TIR from 59% to 63% during the lockdown. The study retrospectively reviewed glucose values downloaded into the cloud [17]. Similarly, a smaller study, including 33 patients with T1D who use the isCGM system that is connected to the clinic, has reported an increase in the TIR from 54% to 65% during the lockdown. However, that study did not indicate whether virtual contact was proposed during the lockdown [18].

We would like to remark on the potential educational role of virtual assistance. Indeed, a training session on the use of the pump and sensor and carbo-counting can be scheduled with specialists including nutritionists or nurses, favoring the access to technologies despite physical distancing [19].

Certain questions regarding teleassistance remain open, including those on cost, reimbursement, authorization, liability, demographic characteristics of the people to be engaged, choice of the method for the virtual visit, involvement of the health care provider, duration of the visit, and adequate time interval between visits [20]. Furthermore, it is important to identify patients who need in-person visits despite the state of emergency, such as the current pandemic. Finally, it is important to consider patients with type 2 diabetes, who represent the majority of patients with diabetes and, in general, have limited access to technology and are less educated in self-managing diabetes. We should probably consider different strategies for type 2 diabetes.

### Limitations

Our prospective study demonstrates the effectiveness of teleassistance in managing disease during the lockdown. Data sharing and remote visits help maintain or achieve adequate glycemic control through data analyses and therapy adjustment. However, our study has some limitations of note. For instance, the patient groups based on different therapeutic strategies are relatively small. Patients with T1D, who constitute the minority of patients with diabetes, frequently adopt personalized insulin delivery schedules and monitoring systems. It is therefore difficult to have large homogeneous patient groups. Furthermore, well-educated patients would have been more likely to provide their consent for virtual visits. This renders our study findings more reliable for patients who have received adequate therapeutic education and are cooperative. Ultimately, the selection criteria of our study were arbitrary; however, they were selected to include the largest number of patients, and we are confident that our findings are potentially applicable to most of our patients with T1D.

### Conclusions

In conclusion, our results show that COVID-19 restrictions have provided an opportunity to bring teleassistance to the frontline in diabetes care. The advancement of technology and the development of new connected devices will further facilitate information exchange among patients, health care providers, and physicians.

## Abbreviations

CGM: continuous glucose monitoring
CSII: continuous subcutaneous insulin infusion
CV: coefficient of variation
DMS: diabetes management system
GMI: glucose management indicator
isCGM: intermittently scanned continuous glucose monitoring
MDI: multiple daily insulin injection
rtCGM: real-time continuous glucose monitoring
SMBG: self-monitoring blood glucose
T1D: type 1 diabetes
TAR: time above range
TBR: time below range
TIR: time in range
